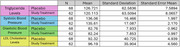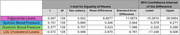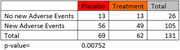# Impact of Icosapent Ethyl on Lipids, Blood Pressures, and Adverse Events in Cognitively Healthy Veterans Participating in the BRAVE Study

**DOI:** 10.1002/alz.094881

**Published:** 2025-01-09

**Authors:** Cecilia A. Cardenas, Carol A. Van Hulle, Hannah Zylstra, Kate Cronin, Aleshia Cole, Elena Beckman, Allison C Eierman, Madeleine Blazel, Karen K Lazar, Kevin M. Johnson, Leonardo A. Rivera‐Rivera, Richard Chapell, Henrik Zetterberg, Carey E. Gleason, Sterling C. Johnson, Sanjay Asthana, Cynthia M. Carlsson

**Affiliations:** ^1^ Division of Geriatrics, Department of Medicine, University of Wisconsin School of Medicine and Public Health, Madison, WI USA; ^2^ Wisconsin Alzheimer’s Disease Research Center, University of Wisconsin School of Medicine and Public Health, Madison, WI USA; ^3^ William S. Middleton Memorial Veterans Hospital, Madison, WI USA; ^4^ Department of Medical Physics, University of Wisconsin‐Madison School of Medicine and Public Health, Madison, WI USA; ^5^ Department of Biostatistics and Medical Informatics, University of Wisconsin School of Medicine and Public Health, Madison, WI USA; ^6^ Department of Psychiatry and Neurochemistry, Institute of Neuroscience and Physiology, The Sahlgrenska Academy, University of Gothenburg, Mölndal, Gothenburg Sweden; ^7^ Clinical Neurochemistry Laboratory, Sahlgrenska University Hospital, Mölndal Sweden; ^8^ Department of Neurodegenerative Disease, UCL Institute of Neurology, London United Kingdom; ^9^ Hong Kong Center for Neurodegenerative Diseases, Clear Water Bay Hong Kong; ^10^ UK Dementia Research Institute at UCL, London United Kingdom; ^11^ Wisconsin Alzheimer’s Institute, University of Wisconsin School of Medicine and Public Health, Madison, WI USA; ^12^ Wisconsin Alzheimer's Disease Research Center, University of Wisconsin School of Medicine and Public Health, Madison, WI USA

## Abstract

**Background:**

The omega‐3 fatty acid eicosapentaenoic acid (EPA) has positive benefits for cardiovascular risk, reducing inflammation and improving endothelial function. Evidence suggests that Icosapent ethyl, a purified form of EPA, can improve cardiovascular outcomes in at‐risk patients. Veterans are at higher risk for vascular dysfunction, a risk factor of Alzheimer’s disease (AD), thus improving vascular health may be pivotal for delaying or preventing AD among Veterans.

**Method:**

Cognitively healthy VA‐eligible Veterans at William S. Middleton Memorial Veteran’s Hospital in Madison, Wisconsin ages 50 through 75 were invited to enroll into an 18‐month randomized, placebo‐controlled, double‐blind, parallel‐group clinical trial which assessed the efficacy of Vascepa® IPE. 206 Veterans were screened for exclusion criteria, leading to the randomization of 131 Veterans to either the placebo or 4mg daily IPE. Triglyceride, low‐density lipoprotein (LDL) cholesterol, and blood pressure were collected at baseline, 9‐months, and 18‐months. New adverse events were self‐reported at all study visits. Lipid levels, blood pressure, and new adverse events were analyzed to determine any differences between Veterans receiving the study treatment or placebo.

**Result:**

Our preliminary analysis indicated no differences for triglycerides, systolic and diastolic blood pressures, and LDL cholesterol between treatment and placebo at baseline (p‐value range: 0.552‐0.880). Cohen’s d values for these ranged from 0.026‐0.108 at baseline. Individuals in the placebo group reported more new adverse events than treatment group (Fisher test = 0.007). Analyses examining if there is any significant change in lipids and blood pressure due to study drug over study duration will be presented at the conference.

**Conclusion:**

Placebo and treatment groups were well balanced in relation to baseline health measures. Study drug was well tolerated. Analyses of study drug efficacy are ongoing.